# Rapid Syphilis Testing Uptake for Female Sex Workers at Sex Venues in Southern China: Implications for Expanding Syphilis Screening

**DOI:** 10.1371/journal.pone.0052579

**Published:** 2012-12-27

**Authors:** Xiang-Sheng Chen, Yue-Ping Yin, Crystal Shen, Guo-Gu Liu, Zheng-Jun Zhu, Wan-Hui Wei, Hong-Chun Wang, Shui-Jie Huang, Jing Li, Joseph D. Tucker, David C. Mabey, Rosanna W. Peeling

**Affiliations:** 1 National Center for Sexually Translated Disease (STD) Control and Chinese Academy of Medical Sciences Institute of Dermatology, Nanjing, China; 2 Mayo Medical School, Rochester, New York, United States of America; 3 Health Bureau of Liuzhou City, Liuzhou, China; 4 Jiangmen Center for STD Control, Jiangmen, China; 5 University of North Carolina School of Medicine, Chapel Hill, North Carolina, United States of America; 6 London School of Hygiene and Tropical Medicine, London, United Kingdom; The Queensland Institute of Medical Research, Australia

## Abstract

**Background:**

Accessibility of syphilis testing services is critical in syphilis control programs for female sex workers (FSWs), but few FSWs attend public STI clinics or other testing sites. Introduction of free rapid syphilis testing (RST) into outreach programs for FSWs will help improve test uptake.

**Methods:**

Commercial sex venues were identified in two cities in South China. In cooperation with health advocacy organizations, health outreach teams from local public health or medical facilities approached all types of sex venues in study areas to offer free RST. Acceptability and uptake of RST among FSWs were evaluated.

**Results:**

A total of 2812 FSWs were offered RST and 2670 (95.0%) accepted syphilis testing. 182 (6.8%) FSWs had a positive RST result among whom 136 (74.7%) were willing to attend an STD clinic for confirmatory testing and treatment. More than half (89, 66.4%) of those with syphilis were not willing to notify their sex partners. Multivariate logistic analysis showed that syphilis test uptake was associated with residing in Jiangmen (AOR, 1.78; 95% CI, 1.15–2.77), older age (AOR, 2.11, 95% CI, 1.17–3.79 for age of 31 years or above), and not working at a service venue (AOR, 1.60; 95% CI, 1.10–2.34).

**Conclusions:**

RST at sex venues is well accepted by FSWs when it is integrated into ongoing outreach services. Such programs provide excellent opportunities for expanding syphilis screening efforts among specific subgroups of FSW who are difficult to reach through clinic-based programs.

## Introduction

Female sex workers (FSWs) in South China have a high prevalence of syphilis and an increasing burden of sexually transmitted HIV infection [1]–[4]. A multi-site survey in China indicates an overall syphilis prevalence of 5.0% among female sex workers (FSWs) but the rates vary significantly among women from different sex work venues [5]. China's most recent national HIV report revealed that HIV cases are increasing among several subgroups of FSWs [6]. Now more than half of all new HIV infections in China are sexually transmitted [7], which has been supported by several molecular epidemiology studies [8]–[10]. Expanding routine STD/HIV testing among China's six million FSWs is an important public health priority [11], but implementing clinic-based STD/HIV screening among FSWs has been challenging [12]. Most FSWs do not attend public STD clinics, or the voluntary testing and counseling (VCT) sites where they can receive free syphilis and HIV testing [13], [14]. FSWs in China may not be aware of their risk of syphilis infection because there are not any specific syphilis screening programs for this population, mistrust of local public health STD clinics about confidentiality and privacy protection, or fear of social stigma and condemnation [15].

On-site syphilis screening using rapid syphilis tests (RSTs) is a potential solution since such simple, rapid, and reliable tests are commercially available [16]. RSTs have been used among FSWs in a number of clinical settings [16], [17], but few studies have reported the use of RST among FSWs at sex venues [18]. Since many FSWs in China do not attend standardized clinics where syphilis testing is available, on-site testing assumes greater potential importance for a community-based approach to testing. The primary goal of this study was to determine RST uptake among FSWs at sex venues in South China.

## Methods

### Study sites

A cross-sectional study was conducted with integration into FSW outreach services at sex work venues in two cities in Southern China provinces (Jiangmen in Guangdong and Liuzhou in Guangxi) during April–August 2009. These two cities were selected to serve as a pilot based on local capacity and the availability of ongoing public health outreach programs focused on FSWs. Prior to this study, the local public health unit identified sex-work venues in each study site. The venues were mapped and selected for recruiting a convenience sample of FSWs for a survey.

### Study participants

FSWs, aged sixteen and older, who were willing to provide verbal informed consent were eligible to participate in the study. For the purposes of this study, only women who had sold sex in the past six months were eligible. Sex-work venues where FSWs solicited clients were categorized into three types, i.e., entertainment venues including karaoke bars, and hotels; service venues including hair salons or barber shops, massage parlors, foot bathing shops, roadside shops, guesthouses, or roadside restaurants; and street-walking venues including streets or public outdoor places.

### Survey design

The survey consisted of a structured questionnaire interview and on-site testing with RST. The questionnaire survey contained 24 items related to socio-demographics, sexual behaviors, willingness to be tested for syphilis, willingness to follow-up at an STD clinic, and willingness to engage in partner notification. The survey was developed in collaboration with a World Health Organization (WHO)-supported project based on discussions with local STD physicians, outreach workers, policy makers, and national syphilis experts.

### Study implementation

Each of the two cities has an FSW outreach team composed of medical professionals (nurses and/or physicians), and public health staff. FSW outreach programs include regular visits to FSW settings to conduct condom promotion, sexual health education, and risk reduction counseling developed according to the national guidelines. Free RST was integrated into these routine outreach services for this pilot study. A total of 218 and 101 venues including walking stands on the streets were reached in Liuzhou and Jiangmen, respectively. Most of the accessed venues accepted the services. As part of this pilot project, the free syphilis testing was offered to all FSWs at sex venues visited by the public health outreach programs. Data regarding sex venues unwilling to participate in the outreach services programs were not collected as part of this investigation. During public health services, eligible participants were taken to a separate, enclosed room to conduct a questionnaire interview and discuss syphilis testing following informed consent. Those who were willing to be tested had whole blood or finger prick blood collected for on-site RST.

### Laboratory testing, clinical diagnosis and treatment procedures

The commercially available treponemal tests (Wantai anti-TP Antibody Rapid Test, Wantai Biological Pharmaceutical Co., Ltd, Beijing, China) were applied as the RST. Using a standard treponemal test (*Treponema pallidum* particle agglutination, TPPA) as reference standard, the sensitivity and specificity of the RST were 95.1% and 95.8%, respectively (Yin YP, unpublished data). The outreach team members were responsible for conducting tests, interpreting the test results, and informing the FSWs of test results on site. Confidentiality of testing results was ensured when the FSWs were informed of their results. All FSWs with positive RST were referred by an outreach team member to designated STD clinics for further diagnosis and treatment according to the national guidelines. In addition, health education and counseling, condom promotion, and partner notification services were provided in the STD clinics. All of the positive specimens and 10% of the negative specimens were sent back to the National Center for Sexually Transmitted Disease Control (NCSTD) in Nanjing for quality control assurance.

### Statistical analyses

The primary outcome of this study was the uptake of syphilis screening, defined by participating in the RST and having the testing result. The secondary outcomes included testing preferences, , willingness to follow up at clinic for confirmatory testing and treatment, and willingness to notify sex partners. To determine univariate relationships, chi-squared (χ^2^) values and unadjusted odds ratio (ORs) with 95% confidence intervals (CIs) were calculated. The variables associated with p<0.10 in univariate analysis were included in the multivariate logistic regression analysis. The final model included variables at level of p≤0.05. Adjusted odds ratios (AOR), with 95% CIs, were calculated. All statistical analyses were performed using SPSS (version 13.0, Chicago, IL) software.

### Ethical review

This study was approved by the Medical Ethics Committee of the Chinese Academy of Medical Sciences Institute of Dermatology and the NCSTD in Nanjing. A verbal informed consent was obtained from all subjects who agreed to participate in the study.

## Results

A total of 2812 women enrolled in this study. Most FSWs were younger than 29 years old, had less than or equal to five sex partners in the previous week, and had no history of prior syphilis infection. Our sample of FSWs included women employed in a variety of sex venues, including 1495 (53.2%), 1156 (41.1%) and 161 (5.7%) from entertainment, service and street-walking venues, respectively. Among 2536 women who responded to question about history of syphilis, 58 (2.3%) had previous syphilis infection. Among those who responded to question of treatment history, 87.4% had the infection treated.

Of the 2812 female sex workers who were offered on-site RST, 2670 accepted testing, giving an overall test uptake rate of 95.0% (95% CI, 94.1–95.7%). Among 2689 FSWs who were willing to get a syphilis testing, 2670 (99.3%, 95% CI 98.9–99.5%) finally got the test. Univariate analysis identified the factors positively correlated with RST uptake, including residing in Jiangmen (OR, 1.97; 95% CI, 1.31–2.97), age of 31 years old or above (OR, 1.79; 95% CI, 1.11–3.15), and not working in a service venue (OR, 1.55; 95% CI, 1.10–2.19). There were not significant differences between women who took test and who were willing but did not take in terms of age, and types of venues. As shown in Table 1, multivariate analysis showed the following factors associated with RST uptake: residing in Jiangmen (AOR, 1.78; 95% CI, 1.15–2.76; P = 0.01), being age of 31 years old or above (AOR, 1.90; 95% CI, 1.02–3.52; P = 0.04), and not working at a service venue (AOR, 1.53; 95% CI, 1.04–2.26; P = 0.03). The following factors were associated with preference of verbal notification of RST results: residing in Liuzhou (AOR, 4.87; 95% CI, 3.82–6.19; P<0.001), working on the street (AOR, 4.14; 95% CI, 2.43–7.06, p<0.001) or at a service venue (AOR, 2.01; 95% CI, 1.55–2.59, p<0.001), and willingness to get RST results immediately on site (AOR, 132.80; 95% CI, 18.34–961.48, P<0.001) The following factors were associated with preference of finger prick blood collection: residing in Jiangmen (AOR, 3.37; 95% CI, 2.71–4.18, P<0.001).

**Table 1:** Multivariate Model Predicting Rapid Syphilis Test (RST) Uptake and Preferences Regarding RST Among FSWs.

Variable	RST uptake		Preference of verbal notification of RST results		Preference of finger prick blood collection	
	AOR (95% CI)	P value	AOR (95% CI)	P value	AOR (95% CI)	P value
Site city						
Liuzhou	Reference	–	4.87 (3.82–6.19)	<0.001	Reference	–
Jiangmen	1.78 (1.15–2.76)	0.01	Reference	–	3.37 (2.71–4.18)	<0.001
Age group (years old)						
20 or below	Reference	–				
21–25	1.05 (0.70–1.58)	0.82				
26–30	1.59 (0.91–2.78)	0.10				
31 or above	1.90 (1.02–3.52)	0.04				
Type of sex work venue						
Street venue	3.10 (0.72–13.36)	0.13	4.14 (2.43–7.06)	<0.001		
Service venue	Reference	–	2.01 (1.55–2.59)	<0.001		
Entertainment venue	1.53 (1.04–2.26)	0.03	Reference	–		
Willingness to get RST results now (on site)						
Yes			132.80 (18.34–961.48)	<0.001	17.95 (10.16–31.69)	<0.001
No			Reference	–	Reference	–

FSWs: female sex workers; AOR: adjusted odds ratio; CI: confidence interval.

A total of 2689 (95.6%) women reported willingness to receive a syphilis test (Table 2). The finger prick method of collecting blood for testing was preferred compared to the standard venous blood draws. Nearly 60% of women reported pain as the reason for not wanting finger-prick blood collection. Most participants preferred being verbally notified of their test results at either the sex venue site (52.5%) or via cell phone (47.0%).

**Table 2:** Preferences Regarding Rapid Syphilis Testing Among FSWs.

Variable	Number (%)
Reports willing to receive syphilis test (n = 2805)	
Yes	2689 (95.6)
No	116 (4.1)
RST Collection Preference (n = 2689)	
Prefer finger prick	1859 (66.1)
Prefer blood draw	830 (29.5)
Reason unwilling to finger prick (n = 326)	
More pain	188 (57.7)
Afraid of infection	61 (18.7)
Affect work	73 (22.4)
Concern of inaccuracy	4 (1.2)
Preferred method of RST result notification (n = 1651)	
Verbal Onsite (sex work venue)	866 (52.5%)
Cellphone	776 (47.0%)
Clinic and Others	9 (0.05%)

FSWs: female sex workers.

Of the FSWs who were tested for syphilis with an on-site RST, 182 (6.8%) had a positive result. Of those with positive RST results, 136 (74.7%) were willing to attend an STD clinic for further confirmatory testing and treatment (Table 3). However, most FSWs (66.4%) with positive RST results were not willing to notify their sexual partners. Among those participants, not being in contact with their sex partner was the most frequent reason provided for their unwillingness to contact their partner (Table 3).

**Table 3:** Follow Up Preferences Among FSWs with RST Positive Results.

Variable	Overall n (%)
**RST result** (n = 2670)	
Negative	2488 (93.2)
Positive	182 (6.8)
**Willing to STD clinic for confirm & treatment** (n = 182)	
Yes	136 (74.7)
No	46 (25.3)
**Preferred time to attend STI clinic** (n = 138)	
Today	38 (27.5)
Tomorrow	26 (18.8)
Two days later	10 (7.3)
One week later	2 (1.5)
When have time	62 (44.9)
**Willing to notify sex partner** (n = 134)	
Yes	45 (33.6)
No	89 (66.4)
**Reason for not willing to notify sex partner** (n = 55)	
Fear of his response	8 (14.6)
Not in contact	25 (45.5)
Separated/divorced	4 (7.3)
Trust him	12 (21.8)
Other	6 (10.9)

## Discussion

Syphilis control programs among FSWs in China are crucial because they have a higher incidence of syphilis and are one of the most-at-risk populations for acquisition and transmission of syphilis and HIV. However, reaching the FSW population, especially certain subgroups, presents various challenges including structural barriers hindering access to care as well as patient's distrust of public clinics [13], [14]. Venue-based introduction of RST can expand testing among these difficult-to-reach FSW subgroups. To our knowledge, this is the first study in China to examine the feasibility of introducing community-based RST among sex workers. A highlight of this study is its diversity of venues, particularly its ability to reach marginalized FSW venues. This study reached women in vulnerable subsets of FSW that are not often included in the current intervention programs for FSWs, such as street-based FSWs or those at massage, sauna and other services venues.

In this study, FSWs had a high overall RST uptake rate (95.1%), which is higher than 57.1% among FSWs in a previous RST study in India [17], or 81.6% among patients at STD clinics a previous study in China [19]. The high up-take rate in our study population is mainly because the RST screening service was integrated into the pre-existing routine FSW outreach services, especially at entertainment venues. Uptake was relatively consistent across venues (and greater than 90%), which reveals the benefit of RST in expanding the reach of testing into those street-walking venues which are usually less covered by the current STD/HIV intervention programs in China. The uptake was also excellent across age ranges, particularly in the older age range who engage in riskier behaviors [2], which is also encouraging as an implication for using RST to expand access to testing.

Correlates of test uptake included delivering RSTs in Jiangmen, older age and not working service venue types. Difference in RST uptake between the two study sites may result from their difference in outreach providers which were the STD clinic outreach teams in Jiangmen but reproductive health hospital or center for disease control outreach teams in Liuzhou. Older FSWs of age 31 years old or above were more than two times as likely to undergo testing. This is consistent with a study of FSWs in China that showed older age associated with increased rates of HIV testing [20]. Older FSWs may have a better perception of risk of infection due to longer history of working as sex workers. FSWs from entertainment or street venues were nearly twice as likely to undergo testing (OR: 1.53, 95% CI: 1.04–2.26). This finding is also consistent with a study of FSWs in China that showed higher tier FSW venues with increased rates of HIV testing [20]. The difference in RST uptake between the FSWs from entertainment- and service-based venues may be related to the higher education background among those in entertainment settings [21]. However, those FSWs who solicited clients on streets are usually poor and marginalized by society and intervention programs. Compared with distribution of health education materials and promotion of condoms, health services including syphilis testing and other reproductive health services are usually more welcome by this population. The relative low-uptake among FSWs in service venues may be due to the lack of trust between these FSWs and the outreach teams. FSWs from service venues typically have decreased opportunities and funds to seek health care, thus may be more likely to utilize an opportunity for free healthcare at their venue.

A high overall willingness to test was stated by FSWs, revealing widespread desire for testing. Of the 6.8% of tested FSWs with positive rapid test results, the majority were willing to go to a STD clinic for confirmation of infection and treatment. The 74.7% rate is slightly higher than a general willingness to attend clinic rate from a previous FSW study in Northern China [13]. Rapid tests can improve clinic attendance due to reaching more FSWs through community-based testing leading to increased subsequent clinic follow-up as FSWs realize the need for care.

FSWs with syphilis were largely unwilling to notify partners. Lack of contact is the most often stated reason, likely due to the often transient nature of client interactions leading to decreased ability for further contact. Although studies have been performed on partner notification in developing countries and China [22]–[24], there is little research on partner notification with FSWs [24]. The 33.6% partner notification willingness rate is consistent with operational studies of partner notification rates in China [22]. However, there are likely differences between the stated willingness for notification and actual notification rate. Developing effective partner notification strategies to overcome these contact challenges could help reduce syphilis transmission.

This study has several limitations. As both study sites are in South China, generalizations to other regions should be made with caution. However, Guangdong Province attracts migrant workers from all over China, and there is much demographic diversity in the study area. The study recruited a convenience sample of FSWs, likely resulting in the sample bias. Also, the rapid syphilis tests have certain limitations as a screening method, particularly among populations with a high prevalence of previous syphilis infection. Hence a positive rapid test result requires a clinic visit for confirmation of syphilis infection. This aspect can be inconvenient and create a barrier for patients [25]. Also, lower sensitivity has been reported with use of point of care rapid syphilis tests [17], [18]. However, the rapid syphilis test has shown overall benefit in improved detection of syphilis, especially in hard–to-reach populations that have not been previously tested and treated [17], [18]. Difficulties may also arise from large-scale implementation of venue-based testing. Evening testing is optimal for reaching many FSWs, but finding outreach workers willing to perform testing during atypical times can be challenging. Despite these limitations, RST has potential as a tool to reach FSWs. A large seven-country study found high acceptability and feasibility for RST among pregnant women in a range of low and middle income settings [26].

Improved rapid syphilis tests are showing promise. A novel point-of-care (POC) syphilis test for the dual detection of nontreponemal and treponemal antibodies has been developed [27]. Screening and confirmatory diagnosis would occur through the same test, reducing the need for clinic visits for confirmatory diagnosis and likely increasing treatment rates. A laboratory-based evaluation of the dual POC has been conducted in multiple sites in China and shown a good performance in sensitivity and specificity as compared with TPPA and TRUST, particularly among specimens with high titer of TRUST [28]. As diagnostic technology continues to advance, the potential for improving syphilis control through rapid syphilis testing continues to grow.

In conclusion, rapid syphilis tests showed high uptake among FSWs in South China. Implementing rapid syphilis testing in sex venues would expand testing among this high risk population, including the subgroup of FSWs on the streets that are more difficult to reach. Rapid syphilis testing would be one important method of improving access to testing and to care within these subgroups, and could play an important role in syphilis control in this high-risk population.
